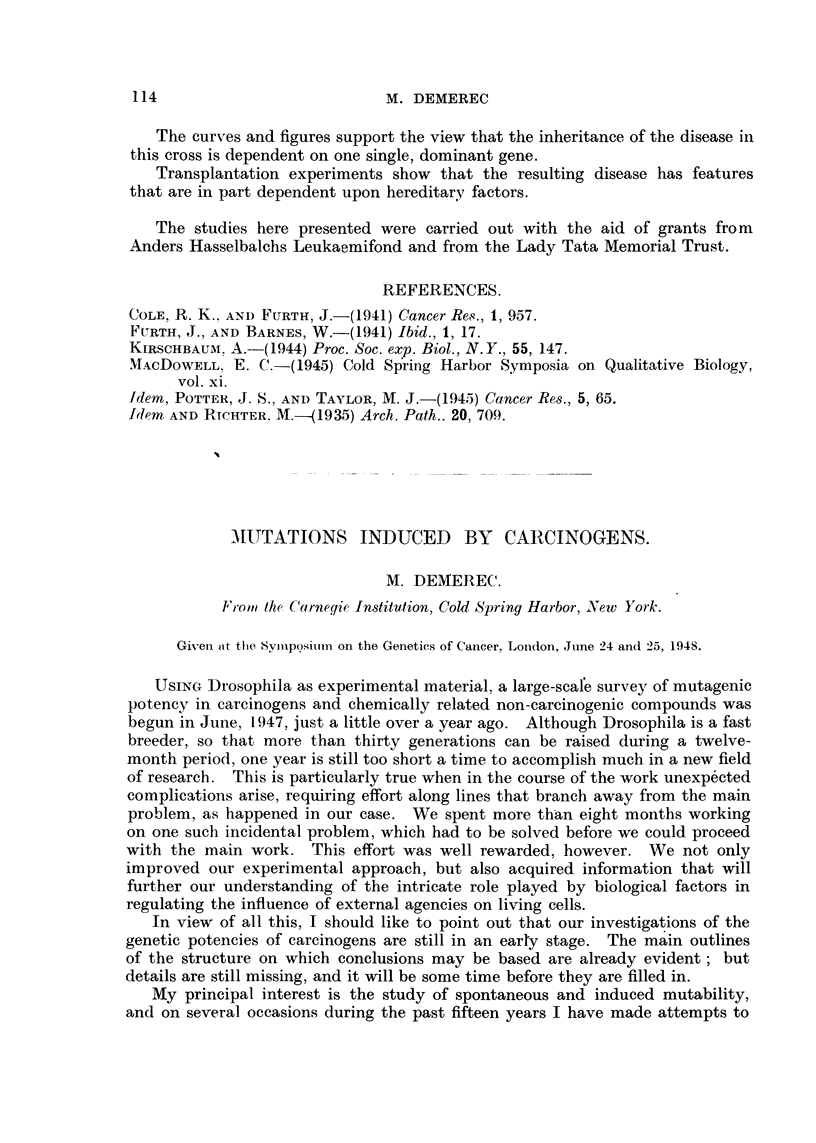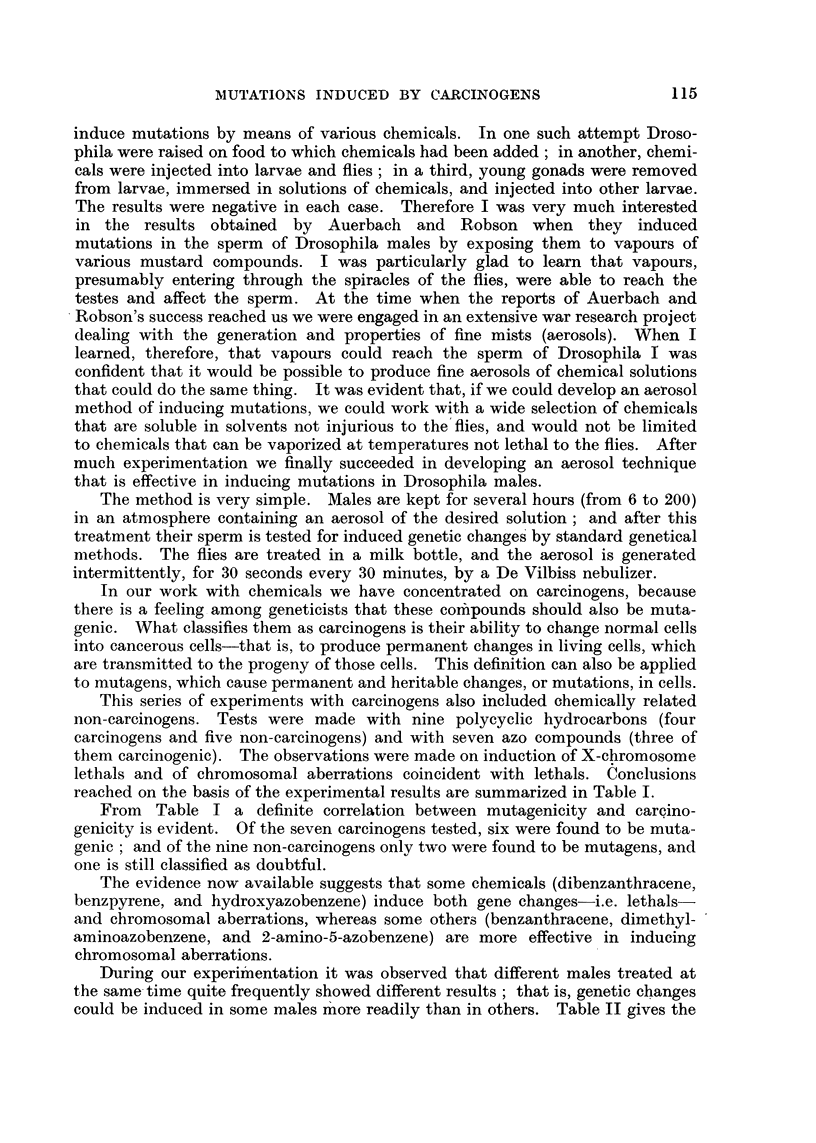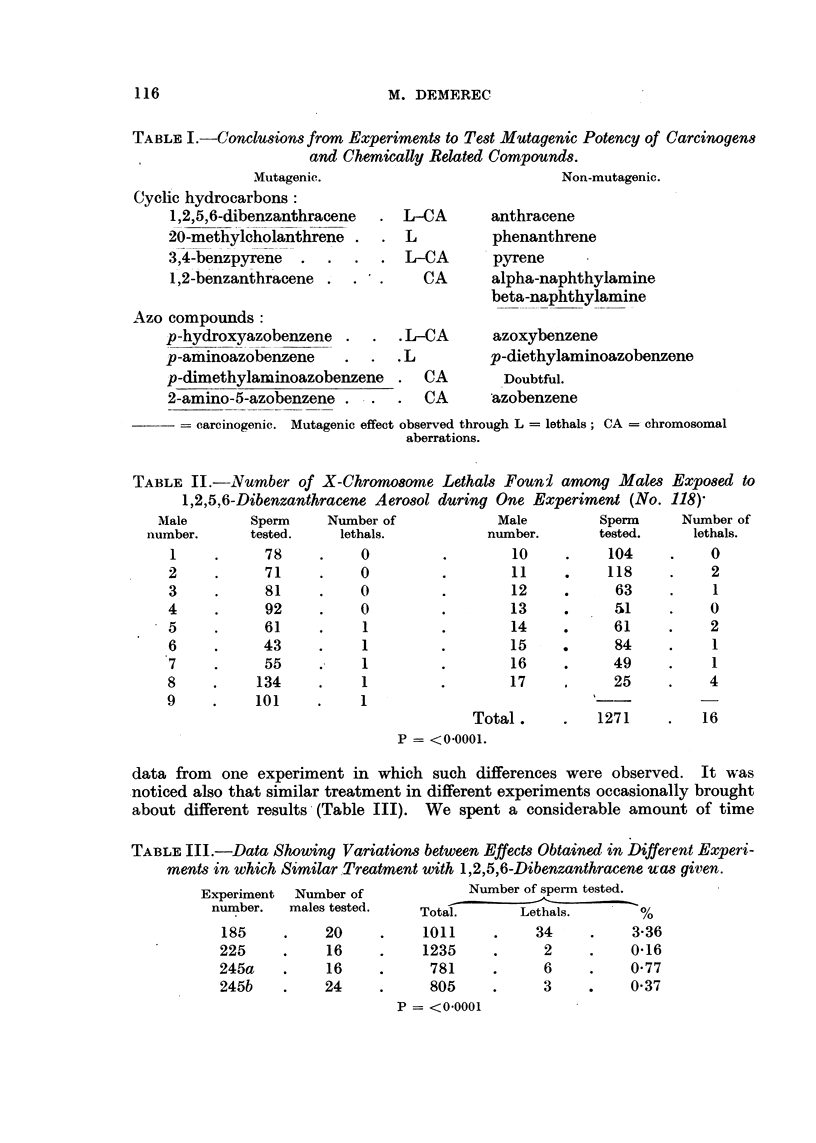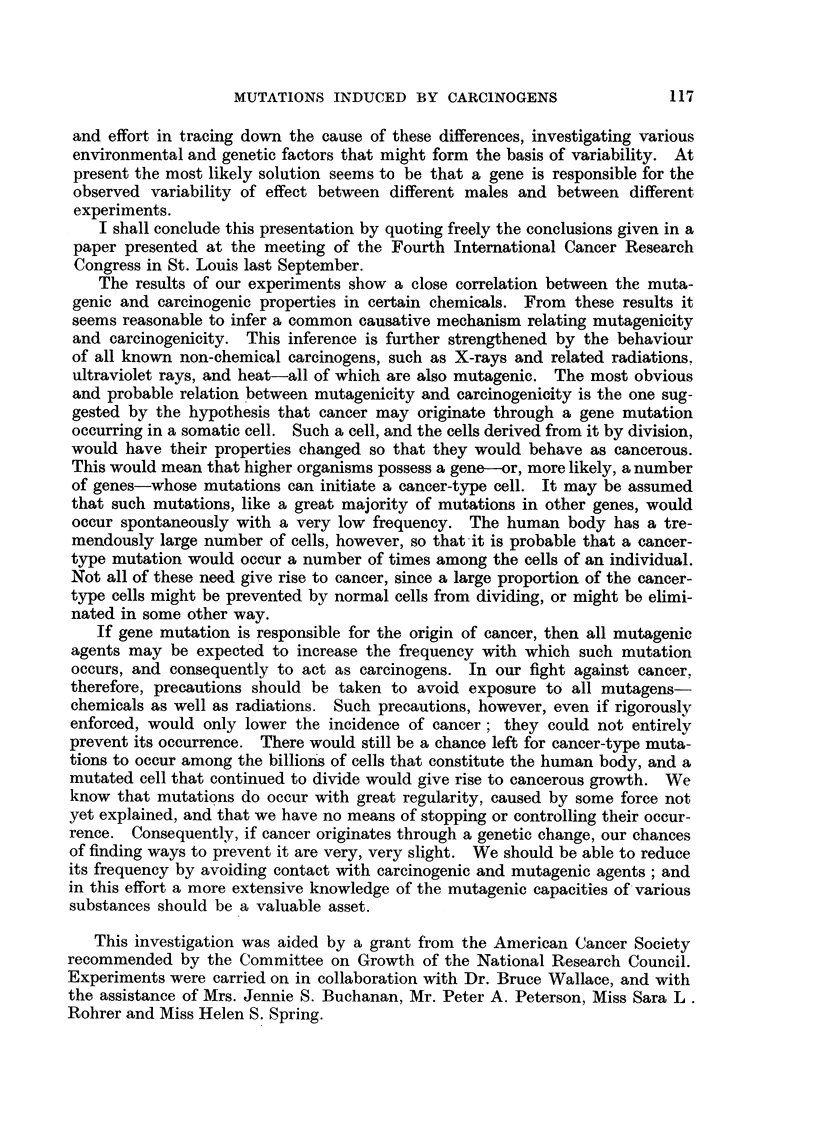# Mutations Induced by Carcinogens

**DOI:** 10.1038/bjc.1948.16

**Published:** 1948-06

**Authors:** M. Demerec


### MUTATIONS INDUCED BY CARCINOGENS.

M. DEMEREC.

*From the Carnegie Institution, Cold Spring Harbor, New York.*

Given at the Symposium on the Genetics of Cancer, London, June 24 and 25, 1948.

Using Drosophila as experimental material, a large-scale survey of mutagenic potency in carcinogens and chemically related non-carcinogenic compounds was begun in June, 1947, just a little over a year ago. Although Drosophila is a fast breeder, so that more than thirty generations can be raised during a twelve-month period, one year is still too short a time to accomplish much in a new field of research. This is particularly true when in the course of the work unexpected complications arise, requiring effort along lines that branch away from the main problem, as happened in our case. We spent more than eight months working on one such incidental problem, which had to be solved before we could proceed with the main work. This effort was well rewarded, however. We not only improved our experimental approach, but also acquired information that will further our understanding of the intricate role played by biological factors in regulating the influence of external agencies on living cells.

In view of all this, I should like to point out that our investigations of the genetic potencies of carcinogens are still in an early stage. The main outlines of the structure on which conclusions may be based are already evident ; but details are still missing, and it will be some time before they are filled in.

My principal interest is the study of spontaneous and induced mutability, and on several occasions during the past fifteen years I have made attempts to induce mutations by means of various chemicals. In one such attempt Drosophila were raised on food to which chemicals had been added ; in another, chemicals were injected into larvae and flies ; in a third, young gonads were removed from larvae, immersed in solutions of chemicals, and injected into other larvae. The results were negative in each case. Therefore I was very much interested in the results obtained by Auerbach and Robson when they induced mutations in the sperm of Drosophila males by exposing them to vapours of various mustard compounds. I was particularly glad to learn that vapours, presumably entering through the spiracles of the flies, were able to reach the testes and affect the sperm. At the time when the reports of Auerbach and Robson's success reached us we were engaged in an extensive war research project dealing with the generation and properties of fine mists (aerosols). When I learned, therefore, that vapours could reach the sperm of Drosophila I was confident that it would be possible to produce fine aerosols of chemical solutions that could do the same thing. It was evident that, if we could develop an aerosol method of inducing mutations, we could work with a wide selection of chemicals that are soluble in solvents not injurious to the flies, and would not be limited to chemicals that can be vaporized at temperatures not lethal to the flies. After much experimentation we finally succeeded in developing an aerosol technique that is effective in inducing mutations in Drosophila males.

The method is very simple. Males are kept for several hours (from 6 to 200) in an atmosphere containing an aerosol of the desired solution ; and after this treatment their sperm is tested for induced genetic changes by standard genetical methods. The flies are treated in a milk bottle, and the aerosol is generated intermittently, for 30 seconds every 30 minutes, by a De Vilbiss nebulizer.

In our work with chemicals we have concentrated on carcinogens, because there is a feeling among geneticists that these compounds should also be mutagenic. What classifies them as carcinogens is their ability to change normal cells into cancerous cells—that is, to produce permanent changes in living cells, which are transmitted to the progeny of those cells. This definition can also be applied to mutagens, which cause permanent and heritable changes, or mutations, in cells.

This series of experiments with carcinogens also included chemically related non-carcinogens. Tests were made with nine polycyclic hydrocarbons (four carcinogens and five non-carcinogens) and with seven azo compounds (three of them carcinogenic). The observations were made on induction of X-chromosome lethals and of chromosomal aberrations coincident with lethals. Conclusions reached on the basis of the experimental results are summarized in Table I.

From Table I a definite correlation between mutagenicity and carcinogenicity is evident. Of the seven carcinogens tested, six were found to be mutagenic ; and of the nine non-carcinogens only two were found to be mutagens, and one is still classified as doubtful.

The evidence now available suggests that some chemicals (dibenzanthracene, benzpyrene, and hydroxyazobenzene) induce both gene changes—i.e. lethals—and chromosomal aberrations, whereas some others (benzanthracene, dimethylaminoazobenzene, and 2-amino-5-azobenzene) are more effective in inducing chromosomal aberrations.

During our experimentation it was observed that different males treated at the same time quite frequently showed different results ; that is, genetic changes could be induced in some males more readily than in others. Table II gives the

TABLE I.—*Conclusions from Experiments to Test Mutagenic Potency of Carcinogens and Chemically Related Compounds.*

| Mutagenic. | | Non-mutagenic. |
|---|---|---|
| Cyclic hydrocarbons : | | |
| 1,2,5,6-dibenzanthracene | L–CA | anthracene |
| 20-methylcholanthrene | L | phenanthrene |
| 3,4-benzpyrene | L–CA | pyrene |
| 1,2-benzanthracene | CA | alpha-naphthylamine |
| | | beta-naphthylamine |
| Azo compounds : | | |
| *p*-hydroxyazobenzene | L–CA | azoxybenzene |
| *p*-aminoazobenzene | L | *p*-diethylaminoazobenzene |
| *p*-dimethylaminoazobenzene | CA | Doubtful. |
| 2-amino-5-azobenzene | CA | azobenzene |

——— = carcinogenic. Mutagenic effect observed through L = lethals ; CA = chromosomal aberrations.

TABLE II.—*Number of X-Chromosome Lethals Found among Males Exposed to* 1,2,5,6-*Dibenzanthracene Aerosol during One Experiment* (*No.* 118).

| Male number. | Sperm tested. | Number of lethals. | Male number. | Sperm tested. | Number of lethals. |
|---|---|---|---|---|---|
| 1 | 78 | 0 | 10 | 104 | 0 |
| 2 | 71 | 0 | 11 | 118 | 2 |
| 3 | 81 | 0 | 12 | 63 | 1 |
| 4 | 92 | 0 | 13 | 51 | 0 |
| 5 | 61 | 1 | 14 | 61 | 2 |
| 6 | 43 | 1 | 15 | 84 | 1 |
| 7 | 55 | 1 | 16 | 49 | 1 |
| 8 | 134 | 1 | 17 | 25 | 4 |
| 9 | 101 | 1 | | | |
| | | | Total . | 1271 | 16 |

P = <0·0001.

data from one experiment in which such differences were observed. It was noticed also that similar treatment in different experiments occasionally brought about different results (Table III). We spent a considerable amount of time

TABLE III.—*Data Showing Variations between Effects Obtained in Different Experiments in which Similar Treatment with* 1,2,5,6-*Dibenzanthracene was given.*

| Experiment number. | Number of males tested. | Number of sperm tested. | | |
|---|---|---|---|---|
| | | Total. | Lethals. | % |
| 185 | 20 | 1011 | 34 | 3·36 |
| 225 | 16 | 1235 | 2 | 0·16 |
| 245*a* | 16 | 781 | 6 | 0·77 |
| 245*b* | 24 | 805 | 3 | 0·37 |

P = <0·0001

and effort in tracing down the cause of these differences, investigating various environmental and genetic factors that might form the basis of variability. At present the most likely solution seems to be that a gene is responsible for the observed variability of effect between different males and between different experiments.

I shall conclude this presentation by quoting freely the conclusions given in a paper presented at the meeting of the Fourth International Cancer Research Congress in St. Louis last September.

The results of our experiments show a close correlation between the mutagenic and carcinogenic properties in certain chemicals. From these results it seems reasonable to infer a common causative mechanism relating mutagenicity and carcinogenicity. This inference is further strengthened by the behaviour of all known non-chemical carcinogens, such as X-rays and related radiations, ultraviolet rays, and heat—all of which are also mutagenic. The most obvious and probable relation between mutagenicity and carcinogenicity is the one suggested by the hypothesis that cancer may originate through a gene mutation occurring in a somatic cell. Such a cell, and the cells derived from it by division, would have their properties changed so that they would behave as cancerous. This would mean that higher organisms possess a gene—or, more likely, a number of genes—whose mutations can initiate a cancer-type cell. It may be assumed that such mutations, like a great majority of mutations in other genes, would occur spontaneously with a very low frequency. The human body has a tremendously large number of cells, however, so that it is probable that a cancer-type mutation would occur a number of times among the cells of an individual. Not all of these need give rise to cancer, since a large proportion of the cancer-type cells might be prevented by normal cells from dividing, or might be eliminated in some other way.

If gene mutation is responsible for the origin of cancer, then all mutagenic agents may be expected to increase the frequency with which such mutation occurs, and consequently to act as carcinogens. In our fight against cancer, therefore, precautions should be taken to avoid exposure to all mutagens—chemicals as well as radiations. Such precautions, however, even if rigorously enforced, would only lower the incidence of cancer; they could not entirely prevent its occurrence. There would still be a chance left for cancer-type mutations to occur among the billions of cells that constitute the human body, and a mutated cell that continued to divide would give rise to cancerous growth. We know that mutations do occur with great regularity, caused by some force not yet explained, and that we have no means of stopping or controlling their occurrence. Consequently, if cancer originates through a genetic change, our chances of finding ways to prevent it are very, very slight. We should be able to reduce its frequency by avoiding contact with carcinogenic and mutagenic agents; and in this effort a more extensive knowledge of the mutagenic capacities of various substances should be a valuable asset.

This investigation was aided by a grant from the American Cancer Society recommended by the Committee on Growth of the National Research Council. Experiments were carried on in collaboration with Dr. Bruce Wallace, and with the assistance of Mrs. Jennie S. Buchanan, Mr. Peter A. Peterson, Miss Sara L. Rohrer and Miss Helen S. Spring.